# Affective Influences on the Intensity of Mental Effort: 25 Years of Programmatic Research

**DOI:** 10.1177/17540739241303506

**Published:** 2025-01-08

**Authors:** Guido H. E. Gendolla

**Affiliations:** Geneva Motivation Lab, FPSE, Section of Psychology, 27212 University of Geneva, Geneva, Switzerland; Swiss Center for Affective Sciences, 27212 University of Geneva, Geneva, Switzerland

**Keywords:** affect, affect priming, effort, cardiovascular response

## Abstract

This article highlights the systematic impact of experienced and implicit affect on the intensity of mental effort. The key argument is that both consciously experienced affect and implicitly activated affect knowledge can influence responses in the cardiovascular system reflecting effort intensity by informing individuals about task demand—the key variable determining resource mobilization. According to the motivational intensity theory, effort rises with experienced demand as long as success is possible and the necessary effort is justified. Twenty-five years of programmatic research have provided clear evidence that both consciously experienced affect and implicitly activated affect knowledge systematically influence the intensity of effort. Importantly, affect's impact on effort is moderated by task context variables, like objective task difficulty, incentive, and other general boundary conditions.

## Resource Conservation and Mobilization

After decades of modest attention to the energetic aspects of human action, current psychology and neuroscience see an increased interest in better understanding resource allocation—with a strong recent focus on the so-called effort-related decision making (e.g., Kool & Botvinick, 2018; Kurzban et al., 2013; Salamone et al., 2017; Shenhav et al., 2017). However, following pioneering work by Ferrero (1894) and Gibson (1900), psychologists have early recognized that effort—the mobilization of resources for action execution (Gendolla & Wright, 2009)—is grounded in a resource conservation principle (e.g., Ach, 1935; Hull, 1943; Tolman, 1932; Zipf, 1949). Resources for action execution are limited, and effort has therefore to be allocated strategically (Kahneman, 1973). Accordingly, organisms try to avoid the waste of effort and thus tend to mobilize just the resources that are necessary for goal attainment, but not more. This fundamental principle simultaneously permits both goal accomplishment and the conservation of limited resources (see Gendolla & Silvestrini, 2015; Richter, 2013, for discussions). Drawing on the resource conservation principle, this article gives an overview of 25 years of programmatic research on affective influences on effort. The reported insights from more than 60 published studies are grounded in what we have learned about the general principles of resource mobilization and its physiological measurement (Richter et al., 2016). It is of note that our research has exclusively focused on exerted or so-called “objective” effort. Exerted and subjectively experienced effort rely at least partly on different processes (Bermúdez & Massin, 2023; Halperin & Vigotsky, 2024) and therefore can be dissociated (e.g., Bijleveld, 2018; Décombe et al., 2022). A possible link between affect and subjectively experienced effort has recently been highlighted by David et al. (2024).

## Basic Principles: Motivational Intensity Theory

Brehm (Brehm, 1975; Brehm et al., 1983; Brehm & Self, 1989) has elaborated the resource conservation principle in a theory that makes precise and fine-graded predictions about how the intensity of effort is determined. Grounded in the principle that people avoid the waste of effort (rather than the effort itself), the motivational intensity theory posits that effort rises with subjective task difficulty as long as success is possible and the necessary effort is justified*.* That is, effort should increase proportionally to the extent of subjective demand until (1) a demand level exceeds a person's abilities (i.e., success is impossible) or (2) the amount of necessary effort is not justified by the magnitude of success importance, which defines the level of *potential motivation*—the hypothetical maximum of justified resources (see Wright, 2008). If one of these limits is attained, effort is predicted to drop sharply to avoid wasting resources. According to this elaboration of the resource conservation principle, subjective demand is the most important variable determining effort and the importance of success (only) sets an upper limit to this relationship. However, under the special condition that task difficulty is not at all clear for an individual, effort can be directly determined by the importance of success.

Motivational intensity theory has offered very clear and easily falsifiable predictions about the basic process of resource mobilization. The next important question is how effort—an input variable for behavior—can be quantified. As discussed in detail elsewhere (Gendolla & Richter, 2010), subjective measures like self-report or output-related measures like task performance are ambiguous. An alternative is assessing effort physiologically during task performance to monitor *activation*, which is—by definition—the key aspect of resource *mobilization*.

## Effort and Cardiovascular Response

To quantify effort intensity, Wright (1996) has integrated Brehm's motivational intensity theory with Obrist's (1981) active coping approach from psychophysiology, resulting in the proposal of a physiological effort intensity measure. According to Wright's integrative analysis, beta-adrenergic sympathetic nervous system impact (reflecting activation) on the heart is proportional to experienced task demand as long as success is possible and the necessary effort is justified. Given that the sympathetic nervous system is responsible for activation and the cardiovascular system is the body's main resource transport system, this measure perfectly fits as operationalization of the effort construct, defined as resource mobilization for action execution (Gendolla & Wright, 2009).

Assessed noninvasively, the beta-adrenergic sympathetic impact becomes manifest in the cardiac pre-ejection period (PEP)—a cardiac contractile force index defined as the time interval between the onset of left ventricular cardiac excitation and the opening of the aortic valve in a cardiac cycle (Berntson et al., 2004). This time interval, which is during rest about 100 ms long, becomes shorter when beta-adrenergic impact increases and is regarded as a reliable index of effort intensity (Albinet et al., 2024; Kelsey, 2012). Cardiac contractile force can also systematically influence other indices of cardiovascular activity, like systolic blood pressure (SBP)—the maximal arterial pressure between two heartbeats (Brownley et al., 2000). This effect of cardiac contractility on SBP is the reason why many earlier studies have used this relatively easy physiological measure as an effort index (our laboratory did not measure PEP before 2007). Both PEP and SBP respond to the level of experienced task demand (e.g., Richter et al., 2008), incentive (e.g., Richter & Gendolla, 2009a), and combinations of both variables (e.g., Silvestrini & Gendolla, 2011a). However, although changes in SBP in the context of task performance are a suitable measure of resource mobilization, PEP is the purer and more sensitive and reliable effort index. Beside cardiac contractile force's impact on SBP via its effect on cardiac output (the volume of blood pumped by the heart), systolic pressure is also determined by peripheral vascular resistance, which is not determined by beta-adrenergic sympathetic nervous system impact. Peripheral resistance's effect on diastolic blood pressure (DBP)—the minimal arterial pressure between two heart beats—is even stronger. HR, another frequently used cardiovascular activity measure, is under both sympathetic (activation) and parasympathetic (deactivation) nervous system control and thus a far noisier effort index than PEP.

Motivational intensity theory's basic predictions have received ample empirical support in more than 150 published studies using cardiovascular measures (see Gendolla et al., 2012b, 2019; Gendolla et al., 2015, part IV; Richter et al., 2016; Wright & Gendolla, 2012; Wright & Kirby, 2001, for reviews). Most relevant for the present discussion, the principles of motivational intensity theory have also been the basis for studying how experienced affect and merely activated affect knowledge can influence effort. I will start with highlighting the role of moods—a prominent and prototypical type of experienced affect.

## Mood, Effort, and Cardiovascular Response

Moods are relatively long-lasting affective states that are experienced without concurrent awareness of their origins (e.g., Schwarz & Clore, 1988, 2007). These characteristics of mood states have important consequences for their potential impact on behavior, which becomes clear by comparing moods with emotions: Experienced emotions are short lived, specific, and object-related (e.g., being happy, sad, and angry *about* something), provide goals (e.g., fear → safety, anger → justice), and comprise physiological adjustments that reflect the mobilization of resources for emotion-specific action (see Behnke et al., 2022; Kreibig, 2010; Mendes, 2016, for overviews). Emotions are “about something” (Arnold, 1960; Lazarus, 1991)—they are organized affective and behavioral reactions to specific internal or external events (Smith & Lazarus, 1990). Experienced emotions have—in contrast to moods—the characteristics of motivational states (Gendolla, 2017). Moods (e.g., feeling down or euphoric) are “feeling states themselves” (Schwarz & Clore, 2007) without a clear motivational function (Martin et al., 1993). Moods themselves neither provide clear action goals, nor do they involve physiological adjustments that reflect the preparation of action—as I will show below. Nevertheless, moods can systematically influence action execution. As I will discuss now, moods can have effects on the magnitudes of subjective task demand and the importance of success—the two variables that explain the principles of resource mobilization in the above-discussed motivational intensity theory (Brehm & Self, 1989).

## The Mood-Behavior-Model (MBM)

The MBM (Gendolla, 2000) posits that moods systematically influence action execution through their *informational* and *directive* impacts. Referring to effort, the informational mood impact influences the level of experienced task *demand*. By contrast, the directive mood impact influences the level of *justified effort—*that is, potential motivation in terms of the above-discussed motivational intensity theory (Brehm & Self, 1989).

The informational mood impact on effort builds on the ample evidence that moods can influence evaluative judgments (see Schwarz & Clore, 2007; Wyer et al., 1999, for overviews). Accordingly, individuals should also use their moods as diagnostic information for behavior-relevant appraisals like “How difficult is the task?,” “Am I able to succeed?,” or “How much do I have to do to accomplish my goal?” Given that effort is grounded in the principle of resource conservation, as discussed above, the subjective impression of task *demand* or *difficulty* should be the most important appraisal for resource mobilization. As for any kind of evaluative judgment, moods can influence such appraisals in a mood congruent manner—people are more optimistic and make more positive judgments in a positive, happy mood than in a negative, sad mood. Consequently, executing a task is experienced as more difficult in a negative mood than in a positive mood—but only as long as the diagnostic, informative value of mood for appraising task demand is not called into question. If people have an external explanation for their current mood, its effect on effort-related responses vanishes (Gendolla & Krüsken, 2002a).

When people face a task, mood is one type of salient information for evaluating task demand. Subjective task difficulty is thus higher in a negative (sad) mood than in a positive (happy) mood. The general result is stronger effort-related cardiovascular reactivity in a negative mood than in a positive mood (e.g., Gendolla et al., 2001; Gendolla & Krüsken, 2001a). However, if objective task difficulty is clear and fixed, mood should have a shifting effect on subjective demand. On each objective difficulty level, task demand should thus appear as higher in a negative mood than in a positive mood. As a result, people in a sad mood should earlier withdraw effort than people in a happy mood. The reason is that an objectively difficult task appears as overchallenging in a sad mood, but as difficult and still feasible in a happy mood.

Besides providing information about task demand, the MBM posits that a directive mood impact refers to the justification of effort for affect-regulative action in compliance with a hedonic motive—approaching feeling good and avoiding feeling bad. Referring to the principles of resource mobilization outlined in the motivational intensity theory (Brehm & Self, 1989), tasks with opportunities for affect regulation should increase the importance of success and thus influence the magnitude of justified effort (i.e., potential motivation). Up to that limit, resources are mobilized in proportion to the level of experienced demand, which can be influenced by the informational mood impact.

**Figure 1.**
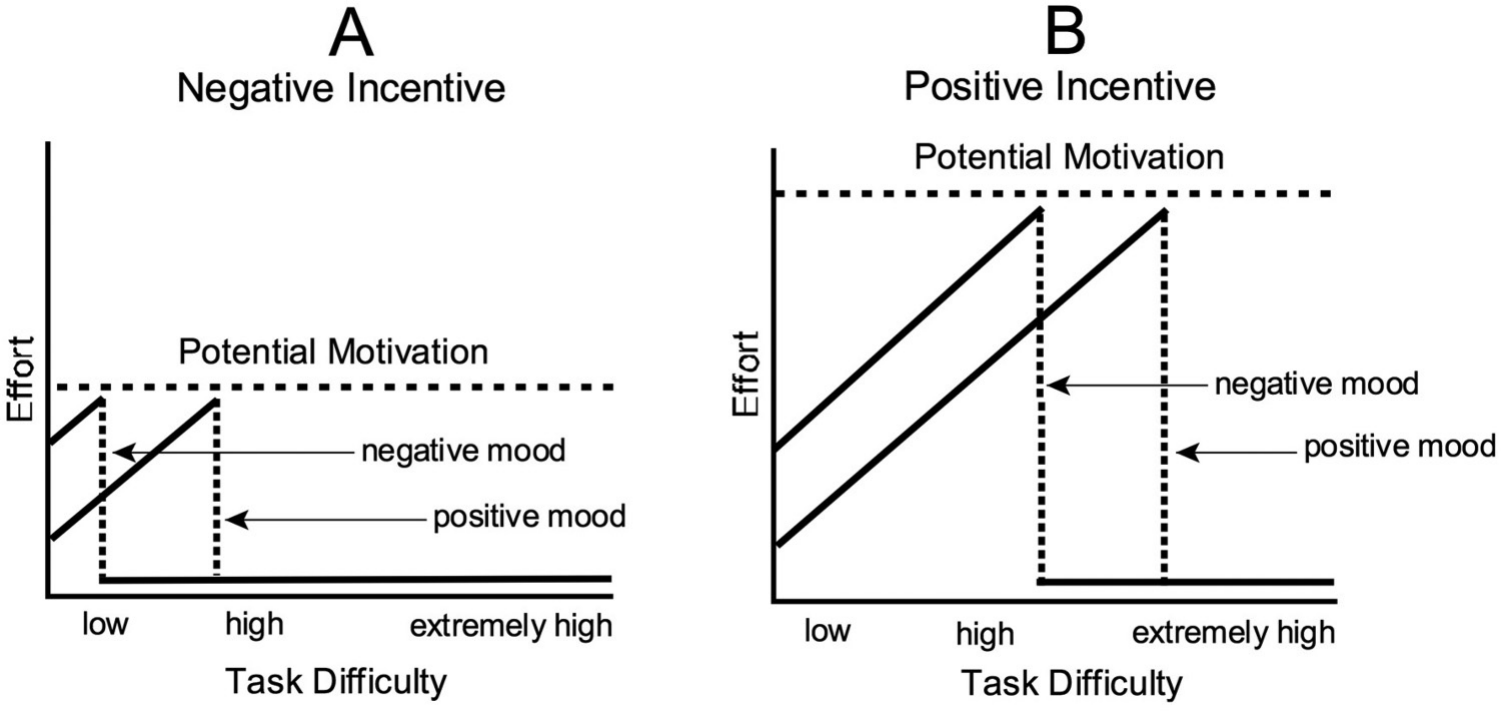
The theoretical interplay of mood, objective task difficulty, and the importance of success (potential motivation) on effort. Panel A shows the impact of mood and task difficulty on effort under the condition of low importance of success (low potential motivation). Panel B shows the effects of mood and task difficulty on effort under the condition of high importance of success (high potential motivation). *Source:* Silvestrini and Gendolla (2009a). Copyright: Elsevier. Reprinted with permission.

The resulting combined effects of mood and success importance under different conditions of objective task difficulty are depicted in Figure 1. Up to the levels of maximally justified effort, mood gives a shift on objective task difficulty's effect on effort.

## Transient Mood Effects on Effort: Empirical Evidence

The MBM predictions have received ample empirical support. The general experimental protocol of this research consisted of (1) a habituation period to assess physiological baseline activity at rest, (2) mood inductions with videos, music, or autobiographical recollection tasks, and (3) a cognitive challenge—typically an attention or memory task. To keep task difficulty stable during the performance period, which is essential for testing effort-related predictions in our theoretical framework, those tasks were relatively short (usually about 5 min). To assess effort, the dependent variable was cardiovascular reactivity during the mood inductions and task performance with reference to the baseline activity values assessed during habituation. Participants were never informed that their affective states should be manipulated. Getting such information reduces mood's diagnostic value for evaluating task demand and makes the mood effect on effort disappear (Gendolla & Krüsken, 2002a). To further disguise the experimental mood manipulations, the mood induction and task performance periods were presented as two independent studies and were separated by some minutes.

Importantly, none of our studies found any mood effects on cardiovascular reactivity during the mood inductions. According to both verbal (mood ratings) and physiological (facial EMG) mood manipulation checks, participants were effectively induced into happy or sad moods, but their cardiovascular systems did not react. The mood manipulations only influenced our cardiovascular effort measures once participants faced a task and could use their mood as diagnostic information for evaluating task demand. This supports the MBM idea that moods themselves are not motivational states, but that they can systematically influence effort—the intensity of motivation—through their informational value for task demand appraisals.

**Figure 2.**
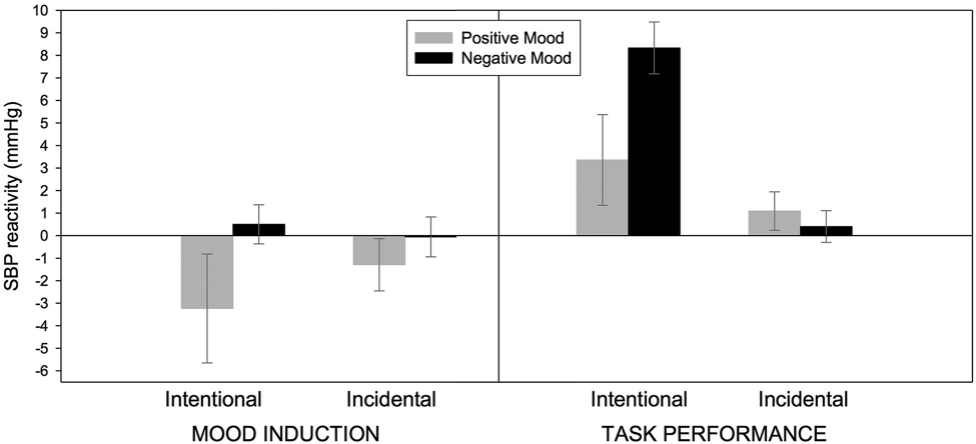
Cell means and standard errors of systolic blood pressure (SBP) reactivity in mmHg in the experiment by de Burgo and Gendolla (2009). Positive values represent higher effort. Copyright: American Psychological Association. Reprinted with permission.

An experiment by de Burgo and Gendolla (2009) illustrates this process. After being induced into happy or sad moods with videos, participants were presented with a list of letter series. In an intentional learning condition, the list presentation was clearly framed as an achievement task—participants were explicitly instructed to correctly memorize all series within 5 min. By contrast, in an incidental learning condition, the list was merely presented for the same time, but nothing was mentioned concerning memorizing or achievement measures. During the mood inductions, SBP reactivity rested on the baseline level and did not differ between the mood conditions, although the verbal mood manipulation checks indicated successfully manipulated mood states. Most relevant, when the letter series list was presented, SBP reactivity was, as expected, stronger in a negative mood than in a positive mood in the intentional learning condition. By contrast, mood had no significant effect in the incidental learning condition. Effects on DBP reactivity mirrored those of systolic reactivity. These results, which are depicted in Figure 2, support the idea that moods only influence effort-related cardiovascular responses when they can be used as task-relevant information for demand appraisals to calibrate effort.

### Context-Dependency: Objective Task Difficulty as a Moderator

The simple mood effect on SBP reactivity was observed in several studies (e.g., Gendolla et al., 2001; Gendolla & Krüsken, 2001a, 2002a, 2002b). However, a negative mood does not always lead to higher effort. Mood's impact is systematically moderated by a powerful task context variable—objective task difficulty. The effects of positive and negative moods for easy and difficult tasks resemble those of high and low ability (see Wright, 1998) and high and low fatigue (see Wright & Stewart, 2012). In contrast to the Mood-as-Information approach (Schwarz & Clore, 1988), which posited that moods should only be used as information according to an “all-or-nothing” principle in global judgments, the MBM relies on the idea that mood is only one piece of information that is integrated with all other available diagnostic information into any judgment (Abele & Petzold, 1994). The underlying process is information integration (Anderson, 1981) rather than misattribution of one's feelings to a judgment object in terms of a “how-do-I-feel-about-it” heuristic (e.g., Schwarz & Clore, 1983). Accordingly, the MBM posits that subjective task demand is determined by both mood and all other diagnostic information like objective task difficulty. This has an important consequence: When people work on a task with a fixed and clear objective difficulty level, they should pragmatically use both types of information—mood and objective task difficulty—to calibrate effort, as demonstrated in a series of experiments (e.g., Gendolla & Krüsken, 2001b, 2002b, 2002c).

In objectively easy tasks, SBP responses were indeed stronger in a negative mood than in a positive mood, because subjective demand and—as a result—effort were both higher in a negative mood. But in objectively difficult tasks, the mood effect on effort was inversed. Here, SBP reactivity was stronger in a positive mood than in a negative mood. The reason for this moderation was that for a difficult task, subjective demand was high but not yet too high in a positive mood, whereas it appeared as overchallenging in a negative mood. However, when objective task difficulty was extremely high, so that succeeding was obviously impossible, the mood could not provide additional diagnostic information. Here, due to disengagement, SBP responses were low in general (Gendolla & Krüsken, 2002c, Study 1).

The experiments discussed above administered attention or memory tasks. Studies by Krüsken (2002, presented in Richter et al., 2006) found corresponding effects with verbal creativity tasks. This is thought-provoking, because usually a positive mood is thought to foster creativity (Isen, 2000), and performance standards are supposed to reduce creativity (Amabile, 1983). However, Krüsken found that a positive mood led to stronger SBP responses and higher creativity performance when task difficulty was unfixed—that is, when participants were asked to do their best. However, when the level of objective task difficulty was manipulated by fixed performance standards (the number of required creative solutions), participants mobilized higher resources and performed better in a negative mood than in a positive mood. Only when task difficulty was objectively very high, the mood effect turned around and SBP responses were again stronger in a positive mood than in a negative mood. That is, mood and performance standards interacted to determine cardiovascular reactivity reflecting effort—also in verbal creativity tasks.

The now discussed evidence that people simultaneously use both their mood and information about objective task difficulty shows that they pragmatically use all available diagnostic information to evaluate the level of task demand. This is reasonable for avoiding the waste of effort in compliance with the principle of resource conservation. However, it is of note that the simultaneous consideration of both mood and objective difficulty contradicts previous theoretical accounts of affective influences on judgments and behavior. Those approaches (Forgas, 1995; Schwarz & Clore, 1988) posited that people *either* heuristically use affect as diagnostic information *or* analytically rely on objective information, like objective task difficulty in the present case. Obviously, this is not what we have found. Moreover, the important replicated finding that mood's impact on cardiovascular response during task performance is moderated by objective task difficulty and that a happy mood consequently leads to higher effort in difficult tasks than a sad mood clearly contradicts accounts positing stable moods effects on resource mobilization in that negative affect should (always) result in higher effort than positive affect (e.g., Morris, 1992; Schwarz, 1990). As we have shown in several studies, mood's impact on effort-related cardiovascular response is highly context-dependent and objective task difficulty is an important moderator of mood's effect on effort. According to our here discussed research, positive and negative moods can lead to low or high effort—in dependence on objective task difficulty. Interestingly, a recent meta-analysis by David et al. (2024) has found that subjectively experienced effort is more simply linked with negative affect. However, in the considered studies, both negative affect and subjectively experienced effort were assessed with respective items of the same instrument—the NASA Task Load Index (Hart, 2006). This limits the evidence to one specific measure. Regarding objective effort and different experimental affect manipulations and measures, the affect-effort link is obviously more complex, though systematic and predictable by considering the principles of motivational intensity theory (Brehm & Self, 1989).

Another context variable moderating moods’ effect on effort is the way people engage in a task—by personal choice or external assignment. In an experiment by Gendolla et al. (2021, Study 2) the personal choice of the type of an upcoming task shielded participants against incidental affective influences on effort intensity during an easy short-term memory task. In contrast to the studies discussed so far, we administered a more subtle mood manipulation during instead of before task performance. However, in accordance with the studies discussed above—which all administered externally assigned tasks—mood inducing sad background music led to stronger cardiac PEP responses during task performance than happy music. This music effect on effort disappeared when participants could ostensibly choose between an attention and a memory task (in fact, all participants worked on the same task that had attention and memory components). Drawing on an elaboration of volition theories (Gollwitzer, 1990; Heckhausen & Gollwitzer, 1987; Kuhl, 1986) in terms of an action shielding model, this was expected because personal task choice should increase commitment and task focus, and thus shield participants against the music effect. This is what we found. This shielding effect against incidental affective stimulation was replicated with a different choice manipulation and a different easy letter counting task (Falk et al., 2022a), and extended in a difficult memory task—in which happy music led to higher effort than sad music when the task was assigned, but not when it was ostensibly personally chosen (Falk et al., 2022b). Two further studies found that personal task choice could also shield effort intensity from the aversive impact of acoustic noise during task performance (Falk et al., 2024b).

### Dispositional Dysphoria Effects

Extending the analysis of affective influences on effort, Brinkmann and colleagues have applied the MBM reasoning about the impact of transient moods to analyze the role of depressive symptoms, especially dispositional dysphoria, in effort (see Brinkmann & Franzen, 2015). Given that a persistent negative mood is a core symptom of depression, Brinkmann and colleagues hypothesized that the impact of depressive symptoms on effort is mediated by mood-congruent appraisals of task demand. Accordingly, dysphoric individuals should evaluate task demand as higher and thus mobilize higher resources for easy to moderately difficult tasks than non-dysphoric individuals. This is what two studies by Brinkmann and Gendolla (2007) found for responses of SBP (Study 1, memory task) and both SBP and HR (Study 2, mental concentration task). Moreover, as for transient mood states, the impact of depressive symptoms on cardiovascular responses was also moderated by objective task difficulty in two studies by Brinkmann and Gendolla (2008) that administered easy versus difficult versions of the previously used memory and mental concentration tasks. That is, for easy tasks, dysphoric individuals showed stronger SBP responses than non-dysphoric individuals. However, due to the increased subjective demand, dysphoric individuals disengaged when objective task difficulty was high, resulting in weak SBP responses. Accordingly, dispositional dysphoria had the same effects as transient mood states. The findings on the impact of depressive symptoms on effort-related cardiovascular response have been replicated and extended by Silvia et al. (2016).

Importantly, the research by Brinkmann and colleagues offers the resolution of a paradox in the literature on depressive symptoms and cardiovascular response. Some studies found that depression is linked to stronger cardiovascular reactivity, while others revealed that it is related to blunted responses (see Salomon et al., 2013; Schwerdtfeger & Rosenkaimer, 2011). As outlined above, considering objective task difficulty as a moderator variable can explain and predict when depressive individuals’ cardiovascular reactivity should be increased and when it should be blunted. Moreover, Brinkmann et al. (2012) found that the effect of dispositional dysphoria on SBP responses could be reversed when participants were made aware that their mood could influence cognitive performance. Likewise, a recent study by Falk et al. (2024a) found that giving dysphoric participants the opportunity to (ostensibly) choose their task themselves attenuated the dysphoria effect on PEP reactivity in an easy short-term memory task. Accordingly, individuals can also control the effect of a dispositional depressive mood on effort, which further advocates for the context-dependency of mood effects on behavior.

In addition to the moderator effect of objective task difficulty, Brinkmann and colleagues have also demonstrated that dysphoric individuals (e.g., Brinkmann & Franzen, 2013; Brinkmann et al., 2009, 2014; Franzen & Brinkmann 2015; Franzen & Brinkmann, 2016a, 2016b; see also Silvia et al., 2014) and patients suffering from clinical depression (e.g., Franzen et al., 2019) are insensitive to reward effects on effort, reflecting anhedonia. While non-dysphoric and non-depressed participants showed stronger responses of SBP, DBP, HR, and PEP if task difficulty was unclear and incentive was high—a condition under which incentive directly determines effort (e.g., Richter & Gendolla, 2006, 2007, 2009a)—dysphoric and depressed individuals did not. This evidence for anhedonia effects suggests that the directive mood impact on behavior, which concerns affect regulation, is impaired by depressive symptoms, while the informational mood impact works well. In individuals who do not suffer from anhedonia, both mood impacts can influence effort, as I will discuss now.

## Mood Effects on Effort for Affect Regulation

Affect regulation is a prominent example of effortful self-regulated action—it requires self-control (Ochsner & Gross, 2005). A study by Silvestrini and Gendolla (2007) investigated the influence of mood on effort exertion in a mood regulation task. Participants were asked to regulate their feelings in terms of attaining the goal of feeling good within five minutes. Predictions were as follows: Based on the informational mood impact, participants in a positive mood should evaluate this task as easier than those in a so-called “neutral” (i.e., actually less intense) mood and especially than those in a negative mood. However, according to the directive mood impact postulated by the MBM, both participants in negative and positive moods should have a stronger need for well-being than those in a neutral mood. Consequently, cardiovascular response during the affect regulation task should be higher in a negative mood (high subjective demand/high justified effort) than in both a neutral mood (relatively high demand/low justified effort) and a positive mood (low subjective demand/high justified effort). After habituation, participants were first induced into a negative, a neutral, or a positive mood through video presentations and then performed the affect regulation task. SBP reactivity described the predicted pattern and was stronger in a negative mood than in both the neutral and positive mood conditions, which were on the same level. The same effect occurred on participants' skin conductance level.

Follow-up studies provided finer-graded analyses of the simultaneous effect of the informational and directive mood impacts on effort, as conceptualized in the MBM. That research was built on the basic idea that actions that are instrumental for hedonic affect regulation—that is, attaining or maintaining the experience of positive affect—justify more effort than actions without such positive hedonic characteristics or outcomes. To test this, these experiments manipulated mood, objective task difficulty, and hedonic incentive simultaneously. In terms of motivational intensity theory (Brehm & Self, 1989), positive hedonic incentive should justify relatively high resources—it makes success important. The actual intensity of mobilized resources should, however, depend on subjective task demand that is jointly determined by objective task difficulty and mood, as discussed above (e.g., Brinkmann & Gendolla, 2008; Gendolla & Krüsken, 2001b, 2002c).

Specific predictions for the combined effect of mood, task difficulty, and hedonic incentive were depicted in Figure 1: When the hedonic incentive of success is low, the magnitude of justified effort is also low. Up to this relatively low level of maximally justified resources, the effort-related cardiovascular response is determined by the informational mood impact: If a task is objectively easy, people in a negative mood mobilize higher resources than people in a positive mood; if a task is objectively difficult, people in a negative mood tend to mobilize little effort. By contrast, when success promises high hedonic incentives, higher resources are justified. Those *justified* resources are, however, only *mobilized* when the level of subjective task demand makes this necessary. This is the case when a negative mood is combined with a difficult task—the condition that leads to disengagement when only low resources are justified. That is, the high hedonic incentive of success should eliminate the effort deficit of people in a negative mood who face a difficult task.

**Figure 3.**
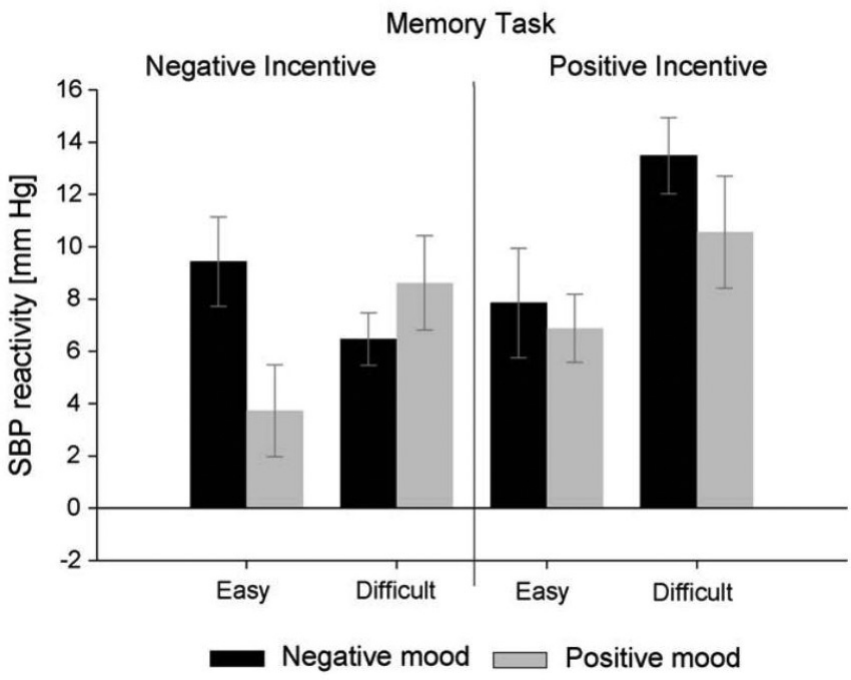
Responses of systolic blood pressure (SBP) in mmHG during the performance on an easy versus difficult memory task in a positive versus a negative mood under conditions of hedonically positive vs. negative incentive in the experiment by Silvestrini and Gendolla (2009a). Positive values represent higher effort. Copyright by Elsevier. Reproduced with permission.

Initial support for the idea that positive hedonic incentive justifies high effort was reported by Gendolla and Krüsken (2002b). In those studies, pleasant performance-contingent consequences of success (listening to elating music) could eliminate the effort deficit of individuals who faced a difficult task in a negative mood: SBP reactivity was the strongest when participants in a negative mood worked on a difficult memory task and expected positive hedonic consequences of success. Another experiment by Silvestrini and Gendolla (2009a) also simultaneously manipulated mood, task difficulty, and hedonic incentive to test the impact of hedonic incentive more directly. After habituation and manipulation of happy vs. sad moods, participants performed either an easy or a difficult memory task with either pleasant or unpleasant consequences of success. In the high-hedonic-incentive condition, participants were promised the presentation of a comedy video in the case of success. By contrast, they expected to see a distressing video after success in the low—in fact, negative—hedonic incentive condition. As depicted in Figure 3, SBP reactivity during task performance described the predicted pattern: When low hedonic incentive was contingent upon success, SBP reactivity revealed the crossover interaction pattern anticipated for the joint effect of mood and objective task difficulty. However, when the hedonic incentive of success was high, the SBP reactivity of participants in the negative-mood/difficult-task condition increased significantly. Here, the positive incentive justified the very high effort that was perceived as necessary. Consequently, effort was very high.

Our results suggest that it is not success per se that justifies the mobilization of high effort. Rather, positive hedonic aspects of success are necessary—the anticipated affective consequences of success define the magnitude of resources that people are willing to mobilize. Corresponding results were found in an experiment that manipulated mood, objective task difficulty, and the pleasantness vs. unpleasantness of a task itself (Gendolla & Silvestrini, 2009b). Task pleasantness had the same effects on SBP reactivity as a positive hedonic incentive in the study by Silvestrini and Gendolla (2009a) discussed above. In addition, responses of HR and DBP largely resembled that effect.

## Summary and Interim Discussion

As presented so far, the basic mechanism for moods’ impact on effort lies in conscious feelings’ informative value for behavior-related judgments in terms of a mood congruency effect (see Schwarz & Clore, 2007; Wyer et al., 1999, for reviews): evaluative judgments are more positive in a happy mood than in a sad mood. That way moods systematically influence appraisals of task demand during performance, which in turn determine effort in accordance with the principles of motivational intensity theory (Brehm & Self, 1989). It is of note that the effects that correspond to those of a transient sad mood and dispositional dysphoria were reported for other types of experienced affect—experimentally manipulated pain (Cancela et al., 2023; Cancela & Silvestrini, 2021) and subjective feelings of fatigue (e.g., Mlynski et al., 2017; Schmidt et al., 2010) and restraint (Mlynski et al., 2021). Moreover, the awareness of mood manipulations and the general task context were identified as boundary conditions of mood's impact on cardiovascular responses reflecting effort (de Burgo & Gendolla, 2009; Gendolla & Krüsken, 2002a).

The so far discussed affect effects on effort are also compatible with recent research highlighting the process underlying resource mobilization for cognitive control, which is necessary for resolving cognitive conflicts (i.e., incompatible response tendencies in one situation). Dreisbach and Fisher (2015) and Inzlicht et al. (2015) have posited that cognitive conflict is aversive and associated with negative affect (e.g., Dreisbach & Fisher, 2012). This should motivate individuals to refocus on goal-directed behavior, recruiting control if necessary (Dignath et al., 2019, 2020), and lead to increased cardiac response reflecting effort in post-conflict task-trials (Spruit et al., 2018). In addition, there is evidence that a sad mood can foster conflict adaptation (van Steenbergen et al., 2010) and that cognitive conflict is—most likely because of its association with negative affect—indeed effortful and leads to increased PEP reactivity in easy cognitive tasks (Bouzidi & Gendolla, 2023a, 2023b, 2024). All this complies with the present reasoning about affect's informative impact on effort. Already previous self-regulation research drawing on cybernetic models conceptualized affect as signaling progress toward goal attainment and driving further behavioral adjustments (e.g., Carver, 2006, 2015; Carver & Scheier, 1990). Overall, this suggests that beside moods, experienced fatigue, and pain also conflict-related affect contributes to behavioral adjustments and effort.

## Implicit Affect and Effort

The next step in our research on affective influences on effort was a radical one: Studying whether merely activating knowledge *about* affective states is sufficient to systematically influence resource mobilization. This research has highlighted the impact of *implicit affect* on behavior. Implicit affect describes the automatic, unintentional activation of individuals’ mental representations of affective states (e.g., Quirin et al., 2009)—without an explicit, conscious affective experience. Implicit affect thus refers to the automatic activation of *emotion concepts* (Niedenthal, 2008)—knowledge about affective states that is stored in long-term memory (Bower, 1981; Lang, 1993; Leventhal, 1982; see also Faul & LaBar, 2023). This knowledge can be unintentionally activated by automatically processed affective stimuli and becomes evident in its impact on behavior (see LeDoux, 1996; Winkielman et al., 2005).

Given that implicit affect does not involve affective experiences, its impact on behavior must happen via a different process than that of moods. Due to their experiential component, moods can function as direct information for evaluative judgments, as discussed above. By contrast, the activation of implicit affect should work according to general principles of knowledge activation—priming. Thus, the effect of affect knowledge on behavior depends on its availability, accessibility, and applicability (see Förster & Liberman, 2007). Keeping with the idea that effort is grounded in a resource conservation principle, especially one feature of individuals’ emotion concepts should be able to systematically influence effort: Performance *ease* or *difficulty* as typical characteristics of different affective states. To explain how this works, I developed the implicit-affect-primes-effort (IAPE) model.

## The IAPE Model

The IAPE model (Gendolla, 2012, 2015) postulates that implicitly processed affective stimuli (e.g., facial expressions, emotion words, etc.) can influence subjective demand and thus effort by rendering information about performance ease or difficulty accessible. Consequently, the mere implicit activation of *knowledge* about affective states is sufficient to influence subjective demand and effort. The conscious experience of the affective *states* themselves is not necessary.

The IAPE model posits that ease and difficulty become available features of emotion concepts through learning. During their life, people acquire knowledge about affective states—they develop emotion concepts (Niedenthal, 2008). Among other characteristics, people learn that coping with challenges is easier in some affective states than in others. Consequently, performance ease and difficulty become available features of the mental representations of those different affective states. Given that effort is grounded in a resource conservation principle, ease and difficulty are highly applicable features when people calibrate their effort to perform a task. The IAPE model posits that making this available and applicable knowledge accessible during task performance leads to experiences of low or high task demand, which in turn determines the resources people mobilize according to the principles of motivational intensity theory (Brehm & Self, 1989).

### Affect-Demand Associations

Considering the above-discussed evidence for experienced sad and happy moods on subjective demand and effort, people should learn that performing tasks is subjectively more demanding in a sad mood than in a happy mood (see Gendolla & Brinkmann, 2005; Gendolla et al., 2012a). That way, ease should become a feature of their mental representation of happiness, while difficulty should turn into a feature of people's sadness concept. These features become accessible by exposing individuals to implicitly processed happiness or sadness stimuli—like very rapidly processed pictures of facial expressions of happiness or sadness—and influence experienced task demand and thus effort.

People should also learn to associate fear with difficulty and anger with ease. Anger, in contrast to fear, is typically linked with high optimism, positive expectations, and experiences of high coping potential (Lerner & Keltner, 2001). High coping potential (or ability), in turn, reduces the level of experienced difficulty during task performance (see Wright, 1998; Wright et al., 2019). Thus, implicit anger should render subjective demand relatively low. The opposite applies to fear: Here, coping potential is typically low (see Lerner & Keltner, 2001; Scherer, 2001; Smith & Lazarus, 1990). Consequently, the implicit activation of the fear concept during task performance should increase subjective demand.

In summary, the IAPE model posits that sadness and fear are associated with difficulty, while happiness and anger are linked to ease. At this point, the IAPE model is explicit regarding the effects of these four types of implicit affect. The theory is, however, not limited to these emotions and can be extended and applied to the representation of any affective state that is associated with ease or difficulty. The IAPE model's central point is that mental representations of affective states that are implicitly activated in the context of task performance systematically influence effort by taking effect on the level of experienced demand. This happens because performance ease or difficulty are features of individuals’ emotion concepts, which can become accessible by the implicit processing of affective stimuli. A series of experiments by Lasauskaite et al. (2017) is of note for this. Those studies applied a sequential priming paradigm and have found the first evidence for automatically activated associative links between implicit affect and ease and difficulty: Implicit sadness was indeed associated with difficulty while implicit happiness was associated with ease.

## Implicit Affect and Effort: Empirical Evidence

In the earlier discussed experiments on the impact of mood—an explicit, consciously experienced affective state—on effort, we first induced participants into happy or sad mood states and let them then work on cognitive tasks. In our research on the impact of implicit affect, we have chosen a more subtle method and activated knowledge about affective states *during* the cognitive tasks. Therefore, participants were exposed to affect primes—briefly flashed and backward masked pictures of facial expressions of emotions--*online* during task performance.

Our first two experiments (Gendolla & Silvestrini, 2011) tested the simple effects of implicitly processed affect primes on cardiovascular responses reflecting effort during an attention and a short-term memory task. To activate implicit affect, briefly flashed (26 ms) backward masked low-resolution front perspective pictures of facial expressions of happiness, sadness, or anger appeared at the beginning of the experimental trials. The affect primes in these and our other studies were taken from the Averaged Karolinska Directed Emotional Faces (AKDEF) database (Lundquist & Litton, 1998). Supporting the IAPE model predictions, both experiments revealed a stronger sympathetic nervous system impact on the cardiovascular system (shortened PEP and increased SBP) in the sadness-prime condition than in both the happiness- and anger-prime conditions. Moreover, there was no evidence for affect prime effects on conscious affect, which was measured before and after the task. However, assessed task appraisals revealed higher subjective demand in the sadness prime condition than in both the implicit anger and happiness conditions. These studies provided the first evidence for the systematic impact of implicit affect on effort as conceptualized in the IAPE model. A follow-up study by Silvestrini and Gendolla (2011b) revealed that the impact of affect primes on cardiovascular response was the strongest if the affect primes did not appear too frequently—ideally in one-third of experimental trials. Therefore, our studies mixed the presentation of affect primes with that of neutral primes, which appeared in two-thirds of the trials.

**Figure 4.**
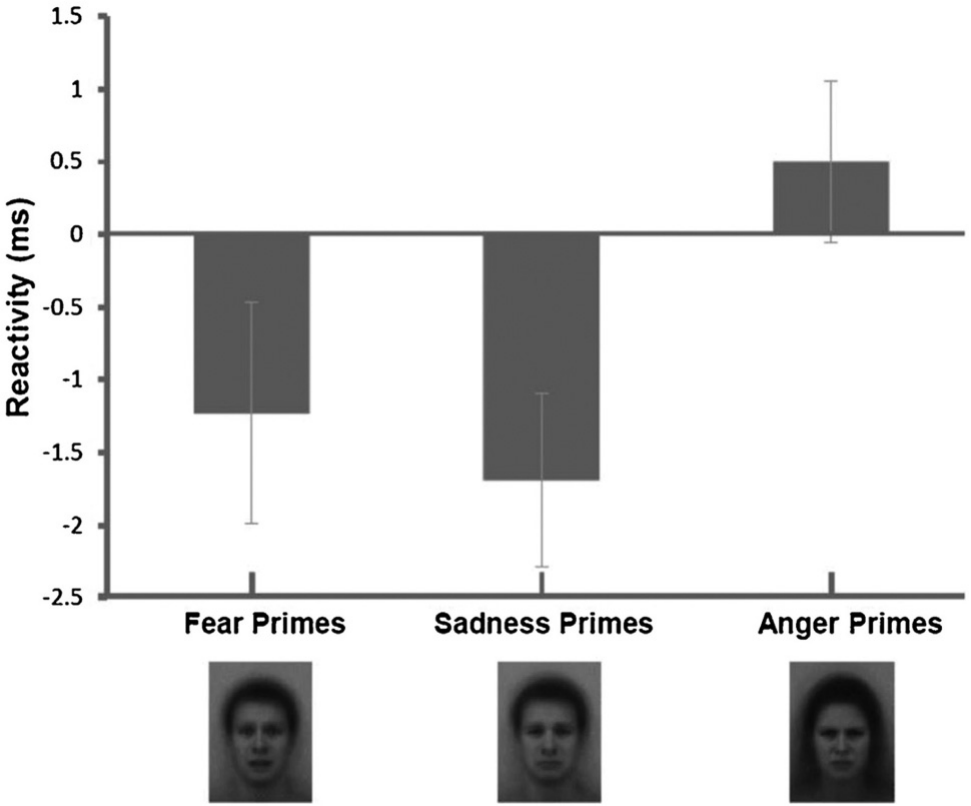
Means and standard errors of responses of the cardiac pre-ejection period (in ms) during task performance in Experiment 2 by Chatelaine and Gendolla (2015). Negative values represent stronger beta-adrenergic sympathetic impact and thus effort. The facial expressions are the affect primes taken from the Averaged Karolinska Directed Faces-AKDEF database (Lundquist & Litton, 1998). Figure Copyright: Elsevier. Reproduced with permission.

Chatelain and Gendolla (2015) extended the evidence for simple implicit affect effects on effort. In one experiment, participants were primed with fear, anger, or happiness during a short-term memory task. Another study exposed participants to fear, anger, or sadness primes during an attention task. Figure 4 depicts the manipulation effects on PEP reactivity. In further support of the IAPE model, both implicit fear and sadness led to higher effort (shortened PEP) than both implicit anger and implicit happiness.

None of our studies has found evidence that the affect primes induced conscious feelings, which in turn could function as direct information for demand appraisals and effort, as discussed earlier. Although this is consistent with the IAPE model idea that implicitly processed affect primes can influence effort without eliciting emotional experiences, it is also to note that zero-effects are hardly convincing. Consequently, Lasauskaite Schüppbach et al. (2013) conducted a more conclusive experiment, which was stimulated by earlier evidence that conscious feelings lose their effect on evaluative judgments when individuals receive a warning that their feelings were manipulated (see Clore, 1992). Under such “warning-conditions,” judgments are usually corrected for the feelings’ influence. Consequently, also consciously experienced affect loses its effect on demand appraisals and effort when participants doubt that their feelings provide valid task-relevant information (e.g., Gendolla & Krüsken, 2002a). In the Lasauskaite Schüppbach et al. (2013) study, participants were primed with happiness or sadness during a moderately difficult attention task. At task onset, half the participants were informed that their feeling state might be manipulated by flickers (actually the primes) that would appear during the task. The study revealed an affect prime main effect: PEP reactivity was stronger in the sadness-prime than in the happiness-prime condition, which is in line with the IAPE model. In addition, there was a warning main effect: PEP reactivity was generally stronger in the warning condition than in the no-warning condition. This latter finding was interpreted as reflecting additional task demand because participants in the warning condition executed two tasks simultaneously. They had to work on the attention task while keeping in mind that the flickers might influence their feelings. However, there was no evidence that the warning manipulation attenuated the effect of the affect primes. That is, apparently, resource mobilization was not piloted by conscious affective feelings whose impact could have been corrected.

### Task Difficulty and Success Incentive as Moderators

As the impact of consciously experienced mood, also implicit affect's impact on effort is moderated by objective task difficulty (Chatelain et al., 2016; Freydefont et al., 2012; Lasauskaite et al., 2014; Silvestrini & Gendolla, 2011c; see also Blanchfield et al., 2014). In objectively easy tasks, sadness and fear primes lead to stronger effort-related cardiovascular responses than happiness and anger primes. However, in objectively difficult tasks, this pattern turns around, and processing anger or happiness primes results in stronger PEP reactivity than sadness or fear primes. The reason is that sadness and fear primes should increase the subjective difficulty of an easy task, resulting in relatively high effort because of high subjective demand. However, these primes should lead to low effort in difficult tasks because of disengagement due to excessive subjective demand. This effect of objective task difficulty should be inverted by happiness or anger primes. Priming happiness or anger in objectively easy tasks should lead to low effort due to low subjective demand. By contrast, effort should be high for an objectively difficult task, because subjective demand should be high but feasible.

**Figure 5.**
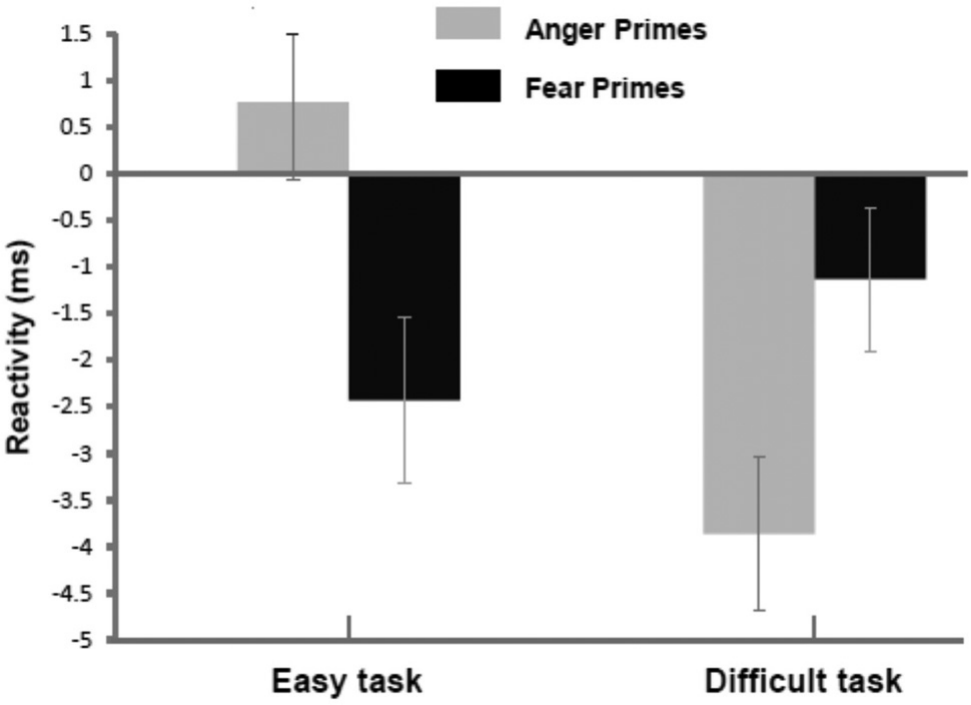
Means and standard errors of responses of the cardiac pre-ejection period (in ms) during task performance in the experiment by Chatelain et al. (2016). Negative values represent stronger beta-adrenergic sympathetic impact and thus effort. Copyright: Elsevier. Reproduced with permission.

Silvestrini and Gendolla (2011c) reported the first evidence for the moderation of implicit affect's effect on effort. Participants were primed with expressions of happiness vs. sadness while they worked on an easy versus difficult version of an attention task. Effects on PEP and SBP showed the expected pattern: Stronger responses in the sadness-easy and happiness-difficult conditions than in the sadness-difficult and happiness-easy conditions. However, the PEP effect was only significant at the beginning of the task. Figure 5 shows the results of an experiment by Chatelain et al. (2016). Participants worked on an objectively easy or difficult mental arithmetic task during which they were primed with fear or anger. As expected, fear primes led to higher effort (shortened PEP) than anger primes when the task was objectively easy. But when the task was objectively difficult, implicit fear led to lower effort than anger. 

Another study by Bouzidi and Gendolla (2024) found that primes of cognitive conflict—pictures of incongruent Stroop task trials (Dreisbach & Fischer, 2012)—has the same effects as primes of sadness and fear: replicating previous findings by Bouzidi and Gendolla (2023a, 2023b), priming cognitive conflict lead to stronger PEP responses in an easy memory task than priming non-conflict (pictures of congruent Stroop task trials). Most relevant, this conflict prime effect was inverted when the memory task was objectively difficult. Here, priming conflict led to weaker PEP responses than priming non-conflict. Accordingly, also cognitive conflict's effect on effort is context dependent—it can lead to relatively high effort in easy tasks, but to low effort reflecting disengagement in objectively difficult tasks.

**Figure 6.**
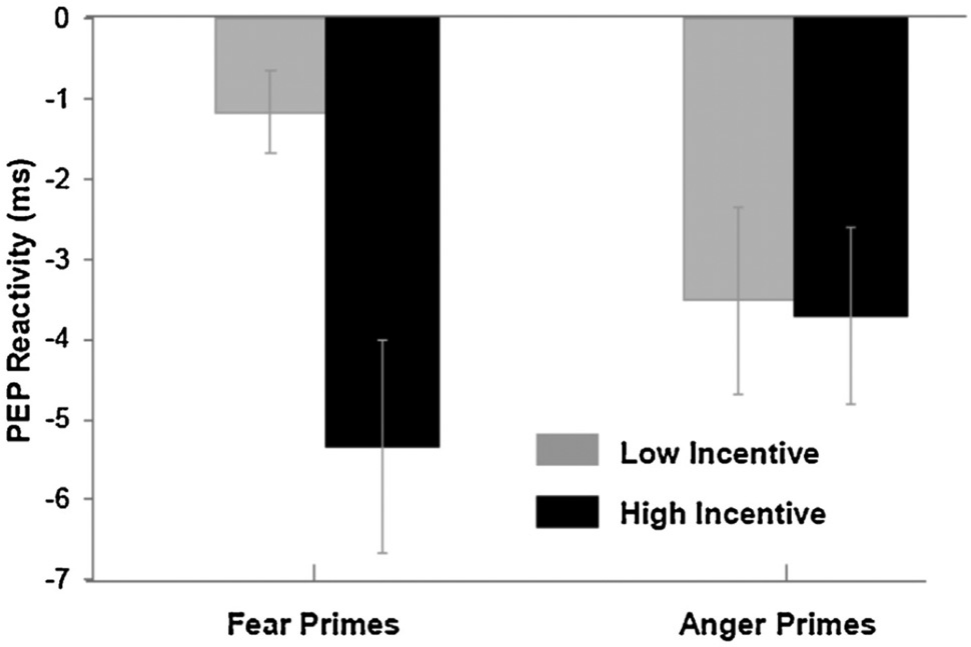
Means and standard errors of responses of the cardiac pre-ejection period (in ms) during task performance in the experiment by Chatelain and Gendolla (2016). Negative values represent stronger beta-adrenergic sympathetic impact and thus effort. Copyright: Elsevier. Reproduced with permission.

Moreover, we found evidence that a high monetary success incentive could eliminate the effort deficit of people working on an objectively difficult task while being primed with sadness or fear (Chatelain & Gendolla, 2016; Freydefont et al., 2012). In compliance with the principles of motivational intensity theory, a high monetary incentive could justify the very high effort that was subjectively necessary when implicit fear or sadness was activated during the performance of an objectively difficult task. Without high incentive, implicit fear and sadness resulted in weak PEP responses, reflecting disengagement in objectively difficult tasks. Figure 6 depicts the PEP results of Chatelaine and Gendolla (2016). Participants worked on an objectively difficult version of a short-term memory task during which they processed primes of fear versus anger. To manipulate success incentive, participants expected a low versus high monetary reward for successful performance. As expected, in the fear prime condition, PEP reactivity was very low when the monetary incentive was low (disengagement), but very high when the incentive was high (very high effort). In the anger prime condition incentive made no significant difference and PEP reactivity fell in between the two fear prime conditions. This was expected because subjective demand should have been high but feasible due to the difficulty buffering effect of implicit anger. Thus, incentive should not make a difference. In addition to the effects on PEP reactivity, corresponding effects occurred on responses of SBP in this study and on HR in the Freydefont and Gendolla (2012) experiment.

Extending the research on implicit affective influences on effort, Silvestrini (2015) found that high monetary incentives also increased PEP, SBP, DBP, and HR responses of participants who implicitly processed pain-related words during a difficult cognitive task. Likewise, Zafeiriou and Gendolla (2017) found a corresponding moderator effect of high monetary incentive on implicitly processed aging primes’ effect on PEP and HR reactivity. Those studies extended the applicability of the IAPE model logic in that they argued that pain and aging are both associated with cognitive performance difficulties (Cancela & Silvestrini, 2021; Zafeiriou & Gendolla, 2018; see also Hess, 2014). Taken together, these effects correspond to those of positive hedonic incentive on resource mobilization in the above-discussed studies on effort for affect regulation (Silvestrini & Gendolla, 2009a, 2009b). 

### Further Boundary Conditions of Implicit Affect's Effect on Effort

The so far discussed affect prime effects on cardiovascular responses reflecting effort depend on the general task context and the unawareness of being primed. Regarding the role of the general task context, an experiment by Framorando and Gendolla (2019a) found that happiness primes only resulted in stronger cardiovascular responses (PEP, SBP, and HR) than sadness primes when the presentation of letter series was framed as a memory task (the task was objectively difficult). This affect prime effect disappeared when the participants were instructed to just watch the letter series. That is, only when participants were in a context that called for effort, the affect primes influenced related adjustments in the cardiovascular system. The primes themselves had no such impact. Implicit affect only influenced effort in a context that permitted accessible ease and difficulty information to be used for evaluating task demand. A corresponding task context effect was discussed earlier for the impact of happy and sad moods on effort (de Burgo & Gendolla, 2009). Moreover, affect primes’ effects on cardiovascular response disappeared (Framorando & Gendolla, 2018a, 2019b) or were even turned into prime-contrast effects (e.g., Framorando & Gendolla, 2018b; Lasauskaite et al., 2014) when participants were made aware of being primed. Accordingly, prime awareness is a general boundary condition for implicit affect's impact on effort.

As for the effects of consciously experienced moods, we also tested whether personal task choice is a boundary condition for implicit affect's effect on effort. As shown for the effects of mood inducing background music (Falk et al. 2022a, 2022b, 2024c; Gendolla et al., 2021) or dispositional dysphoria (Falk et al., 2024a), also affect primes lose their effect on effort when participants can choose the type of tasks or task characteristics like the color or the typeface of task stimuli themselves (Framorando et al., 2023, 2024a, 2024b; see also Bouzidi & Gendolla, 2023a). The same shielding effect was found in individuals with a high dispositional action orientation (Bouzidi & Gendolla, 2023b).

## Summary and Interim Discussion

Summing up, the studies discussed in this section have revealed replicated empirical evidence for implicit affect's systematic impact on effort as conceptualized in the IAPE model (Gendolla, 2012, 2015). We have found evidence for simple affect prime effects on cardiovascular responses reflecting effort and for the moderation of these effects by objective task difficulty and performance-contingent incentives. The first two moderator effects followed the logic of motivational intensity theory by influencing subjective demand and the level of justified effort. The effects of sadness and fear primes have been conceptually replicated for stimuli that implicitly activate mental representations of pain and aging. Moreover, prime awareness, prime adaptation, the general task context, and personal task choice were identified as boundary conditions of implicit affect's impact on cardiovascular responses reflecting effort.

## Affect and Effort: Conclusions and Outlook

This article has given an overview of 25 years of programmatic research on affective influences on the intensity of effort assessed as responses in the cardiovascular system. That research was based on two different theories about the impact of consciously experienced affect (the MBM; Gendolla, 2000) and implicit affect (the IAPE model; Gendolla, 2012) on human action. Grounded in the principles of motivational intensity theory (Brehm & Self, 1989), these studies have revealed ample replicated evidence that affect primarily influences resource mobilization by informing about subjective task demand. People rely on affect to guide their behavior according to the resource conservation principle (Ach, 1935; Ferero, 1894; Gibson, 1900; Hull, 1943; Tolman, 1932; Zipf, 1949). Importantly, in contrast to other interpretations of the resource conservation principle (e.g., David et al., 2024; Inzlicht et al., 2018; Kool et al., 2010, to name a few) this does mean that people avoid effort itself. If high effort is necessary and justified, people are obviously fine with mobilizing high resources. What people try to avoid is *wasting* effort (Brehm & Self, 1989). That is, effort follows the same principles that apply to the use of a different resource—money. People do not avoid money and people do also not avoid spending money. What people usually try to avoid is wasting money—they do not want to pay higher prices than necessary and justified. For the present analysis, the important point is that affect helps to calibrate resource mobilization by informing about task demand and thus the amount of effort that should be mobilized.

Interestingly, Aarts, Custers, and colleagues (see Custers & Aarts, 2005, 2010 for overviews) have reported replicated evidence that implicitly processed positive affective stimuli can augment individuals’ performance and have argued that implicit effects on effort are the central mechanism behind this effect. At first glance, this seems to contrast with our replicated finding that negative affect (sad mood, experienced and primed pain, implicit sadness, implicit fear, primed cognitive conflict) can result in higher effort than positive affect (e.g., Bouizidi & Gendolla, 2023a, 2023b; Cancela et al., 2023; Chatelain & Gendolla, 2015; Gendolla et al., 2001; Gendolla & Silvestrini, 2011; Silvestrini, 2015). However, we have also found that affect's influence on effort turns around if objective task difficulty is high. Here, a happy mood or implicit happiness result in stronger cardiovascular responses reflecting effort than a sad mood or implicit sadness (e.g., Gendolla & Krüsken, 2001b; Silvestrini & Gendolla, 2011c). Thus, assuming that objective task demand in the Aarts and Custer group's studies was relatively high (which is the default in most studies on cognitive performance) offers an easy way to reconcile their findings with ours—under the premise that the performance effects reported by Aarts, Custers, and colleagues are indeed the outcome of effort exertion. Positive affective stimuli could have reduced subjective demand leading to higher effort than negative affective stimuli that should have rendered subjective demand high, leading to disengagement. Moreover, the positive affective stimuli in the studies by Aarts, Custers, and colleagues could have increased the level of justified effort, as in our studies on affect regulation, justifying the mobilization of higher resources if necessary.

## Effort and Performance

The studies discussed in this overview were designed for measuring the intensity of effort physiologically. However, as in the research by Aarts, Custers, and colleagues, all of our studies also assessed task performance, but given our study designs and protocols, we did so without hypotheses. The reason is that cognitive psychology usually explains performance by capacity rather than effort. Moreover, it is hard to predict how effort should increase performance in our frequently administered objectively easy tasks, in which success is possible with little effort. However, some of the here discussed studies found corresponding effects on both cardiovascular responses and performance outcomes (e.g., Framorando & Gendolla, 2018a; Gendolla & Silvestrini, 2011, Study 1; see also Framorando & Gendolla, 2023), some found correlations between both measures (e.g., Gendolla et al., 2001; Gendolla & Krüsken, 2002b, 2002c, Study 1; Lasauskaite Schüppbach et al., 2013), while most others found only the predicted effects on effort intensity. However, this is not surprising for conceptual and methodological reasons—the effort-performance link is multifaceted (Halperin & Vigotsky, 2024).

Conceptually, effort and performance are not interchangeable constructs. Effort refers to the mobilization of resources for action execution (Gendolla & Wright, 2009) whereas performance describes (only) the outcome of instrumental behavior—and several quantifications of that outcome are possible. Beyond mere quantitative aspects of performance (e.g., speed or force) it has been proposed to distinguish between effectiveness (i.e., accuracy) and efficiency (i.e., effectiveness in relation to effort) to measure performance (e.g., Eysenck & Calvo, 1992). Moreover, it was shown that people with high task-related abilities can perform well with only little effort, whereas people with lower abilities must expend high effort without any guarantee that this will bring return (see Wright, 1998; Wright & Mlynski, 2019). Effort is often exerted to compensate for ability deficits (Hockey, 1997) with the result that higher effort in persons with low ability results in the same performance as little effort in individuals with higher ability (e.g., Smith & Hess, 2015). This does not mean that effort is not linked with performance. It is, however, only one factor. Performance is beside effort determined by capacity, persistence, and strategy use (Locke & Latham, 1990). Consequently, performance effects should be interpreted with caution rather than assuming that speed and accuracy are the direct effect of the intensity of effort or that performance effects are valid and reliable indicators of effort intensity.

On the methodological level, it is of note that studies focusing on cognitive performance outcomes are usually run in within-persons designs with dozens or hundreds of task trials to account for large individual differences in response speed and accuracy. The here discussed studies were designed to assess the impact of affect on physiological indicators of effort intensity. This called for between-persons designs and relatively short tasks with well-defined and stable difficulty levels and with a minimal risk for inducing affective states themselves. We wanted to prevent changes in subjective difficulty due to learning, fatigue, or boredom effects. For that reason, participants did only receive performance feedback in training trials, but not in the main tasks. Feedback is beneficial for performance (Locke & Latham, 1990), but it also induces affective reactions (e.g., Kreibig, 2010; see also Carver, 2006) that could interfere with our affect manipulations. Taking all that into account, the here discussed research permits clear conclusions about the role of affect for effort intensity, but not about links between effort and performance. Specifying this link is a challenge for future research.

## Effort Decisions—An Alternative?

According to the present analysis, the level of subjective demand is the most important variable influencing effort. However, it should also be noted that there are recent effort decision models (e.g., Kurzban et al., 2013; Shenhav et al., 2017; see Kool & Botvinick., 2018, for an overview) that focus on persons' mental representations of costs and benefits associated with task performance and frequently neglect the decisive role of subjective demand. In these models, effort is the outcome of cost/benefit calculations. Effort decision models aim to model individuals’ estimates of the value of allocating resources to a given task and their selection of the action with the highest expected value. This selection process is then linked to the intensity of a particular action (e.g., how much cognitive control one is willing to allocate to a task).

It is possible to integrate effort decision models with the principles of motivational intensity theory (see Silvestrini, 2017; Silvestrini et al., 2023). However, effort decision models have been primarily built to explain choice behavior—effort-related decisions underlying cognitive task performance (but also animal behavior, Salamone et al., 2017)--that recruits executive functions with links to relevant brain areas. Their primary goal is not to explain how the intensity aspect of action—i.e., resource mobilization itself—is determined (see Gendolla & Richter, 2013; Silvestrini & Gendolla, 2019). As presented in this overview, motivational intensity theory offers clear and precise predictions about resource mobilization itself and how to measure it (Wright, 1996). Moreover, this theory’s predictions about effort are supported by more than 150 published studies that have tested many variables’ effects on effort in widely varying contexts (see Gendolla et al., 2012b, 2019; Richter et al., 2016; Wright & Kirby, 2001)—the here discussed more than 60 studies on affective influences on effort intensity are only a part of that. In light of this evidence, one may wonder whether behavioral choices between effortless and effortful action alternatives (e.g., Inzlicht et al., 2018; Kool & Botvinick, 2018; Kurzban et al., 2013; Shenhav et al., 2017) can inform about processes underlying actual resource mobilization. Usually, this is not directly measured in effort-based decision-making research and, as discussed above, task performance measures face many challenges as effort index.

The decision to engage in an action or to disengage may depend on the required (i.e., anticipated) or actual effort. Accordingly, effort-based decision-making and actual resource mobilization may interact with each other (e.g., Manohar et al., 2015). One possibility is that effort-based decision making determines the level of justified effort (i.e., potential motivation) rather than effort intensity itself (Gendolla & Richter, 2013). Considering these issues provides good reasons for assessing effort directly in accordance with its definition as the mobilization of resources for action execution (Gendolla & Wright, 2009) and to investigate effort in a theoretical framework that permits clear hypotheses about resource mobilization by clarifying how its central predictor variables function and interact. Motivational intensity theory (Brehm & Self, 1989) and its integration with research and insights from psychophysiology (Wright, 1996) has offered all this decades ago—and as discussed in this overview, affect plays a decisive role in this process by informing about task demand.

## The Important Role of the General Task Context

An issue that merits more attention in future research on affective influences on effort are the roles of the general task context. Richter (2010) could show that it largely depends on the general task framing which variables take effect on effort-related cardiovascular reactivity. If participants had to rate subjective task difficulty before performing a task, cardiovascular reactivity was a function of difficulty. However, if participants had to rate the significance of a promised reward instead, cardiovascular reactivity was determined by the reward value. Apparently, the general task framing can determine which information individuals use for resource mobilization. This should also have implications for affective influences on effort.

The here discussed research highlighted the important role of subjective demand in resource mobilization. However, individuals’ affective state can also inform about the instrumentality of success and thus influence potential motivation—the level of justified effort. This was shown in an experiment by Richter and Gendolla (2009b). After being induced into a positive, neutral, or negative mood, participant worked on a memory task of unclear task difficulty—a setting in which participants (have to) orient resource mobilization on potential motivation, as discussed above. Before the task, participants rated the probability of winning a monetary reward in the case of success. Participants in a happy mood were more optimistic to win than those in a sad mood—suggesting that potential motivation was higher in a positive mood (high probability to get the reward) than in a negative mood (low probability to get the reward). Corresponding to this, SBP reactivity during task performance linearly increased from the negative via the neutral to the positive mood condition.

Other recent studies demonstrated that affective influences on effort can be moderated by the reason why people engage in an action—personal action choice vs. external action assignment. The personal choice of tasks or task characteristics can lead to shielding against the effects of mood inducing music (Falk et al., 2022a, 2022b, 2024c; Gendolla et al., 2021), dispositional dysphoria (Falk et al., 2024a), affect primes (Framorando et al., 2023, 2024a, 2024b), primed cognitive conflict (Bouzid & Gendolla, 2023a, 2023b, 2024), and aversive acoustic noise (Falk et al., 2024) on effort. That is, affective influences on effort may be reserved for performance settings in which commitment and task focus are relatively low, as it is frequently the case in externally assigned tasks. 

## Coda

As presented in this overview of more than 60 studies conducted in 25 years of programmatic research, both experienced and implicit affect can systematically influence the intensity of effort—the mobilization of resources for action execution. This work contributes to the many other studies that have shown that effort primarily relies on the principle of resource conservation. The elaboration of this principle in terms of motivational intensity theory and its extensions to explain the role of affect brought structure into the understanding of how affect can influence the intensity aspect of action execution. Importantly, that research could not only provide well-replicated evidence that affect influences effort. It has also identified important moderator variables—like objective task difficulty, performance-contingent incentive, the general task context, awareness of affective influences, and personal task choice—in this process and it has identified general task context variables, like the way people engage in action. Especially these recent findings open the door to the next decade of research on affect and effort.
